# Supplementary material for: Medical negligence claims and the health and life satisfaction of Australian doctors: a prospective cohort analysis of the MABEL survey
Source: BMJ Open. 2022 May 19;12(5):e059447. doi: 10.1136/bmjopen-2021-059447 (PMC9121477; doi:10.1136/bmjopen-2021-059447)
Supplement: Author's manuscript [file bmjopen-2021-059447.draft_revisions.pdf]

BMJ Open is committed to open peer review. As part of this commitment we make the peer review history of every article we publish publicly available.

When an article is published we post the peer reviewers' comments and the authors' responses online. We also post the versions of the paper that were used during peer review. These are the versions that the peer review comments apply to.

The versions of the paper that follow are the versions that were submitted during the peer review process. They are not the versions of record or the final published versions. They should not be cited or distributed as the published version of this manuscript.

BMJ Open is an open access journal and the full, final, typeset and author-corrected version of record of the manuscript is available on our site with no access controls, subscription charges or pay-per-view fees (http://bmjopen.bmj.com).

If you have any questions on BMJ Open's open peer review process please email info.bmjopen@bmj.com

BMJ Open

Medical negligence claims and the health and life satisfaction of doctors: a prospective cohort analysis of the Australian MABEL survey

Journal:	*BMJ Open*
Manuscript ID	bmjopen-2021-059447
Article Type:	Original research
Date Submitted by the Author:	22-Nov-2021
Complete List of Authors:	Bradfield, Owen; The University of Melbourne, Melbourne School of Population and Global Health;
Keywords:	MENTAL HEALTH, Health & safety < HEALTH SERVICES ADMINISTRATION & MANAGEMENT, Health policy < HEALTH SERVICES ADMINISTRATION & MANAGEMENT, Quality in health care < HEALTH SERVICES ADMINISTRATION & MANAGEMENT, MEDICAL LAW, PUBLIC HEALTH

SCHOLARONE™
Manuscripts

I, the Submitting Author has the right to grant and does grant on behalf of all authors of the Work (as defined in the below author licence), an exclusive licence and/or a non-exclusive licence for contributions from authors who are: i) UK Crown employees; ii) where BMJ has agreed a CC-BY licence shall apply, and/or iii) in accordance with the terms applicable for US Federal Government officers or employees acting as part of their official duties; on a worldwide, perpetual, irrevocable, royalty-free basis to BMJ Publishing Group Ltd ("BMJ") its licensees and where the relevant Journal is co-owned by BMJ to the co-owners of the Journal, to publish the Work in this journal and any other BMJ products and to exploit all rights, as set out in our licence.

The Submitting Author accepts and understands that any supply made under these terms is made by BMJ to the Submitting Author unless you are acting as an employee on behalf of your employer or a postgraduate student of an affiliated institution which is paying any applicable article publishing charge ("APC") for Open Access articles. Where the Submitting Author wishes to make the Work available on an Open Access basis (and intends to pay the relevant APC), the terms of reuse of such Open Access shall be governed by a Creative Commons licence – details of these licences and which Creative Commons licence will apply to this Work are set out in our licence referred to above.

Other than as permitted in any relevant BMJ Author's Self Archiving Policies, I confirm this Work has not been accepted for publication elsewhere, is not being considered for publication elsewhere and does not duplicate material already published. I confirm all authors consent to publication of this Work and authorise the granting of this licence.

For peer review only

ARTICLE TYPE	Original research
TITLE	**Medical negligence claims and the health and life satisfaction of doctors: a prospective cohort analysis of the Australian MABEL survey.**
Authors	Dr Owen Bradfield[1] (owenmb@student.unimelb.edu.au)<br>A/Prof Marie Bismark[1] (mbismark@unimelb.edu.au)<br>Prof Anthony Scott[2] (a.scott@unimelb.edu.au)<br>A/Prof Matthew Spittal[3] (m.spittal@unimelb.edu.au)<br><br>[1] Law and Public Health Unit, Centre for Health Policy, Melbourne School of Population and Global Health, University of Melbourne, Australia.<br>[2] Melbourne Institute of Applied Economic and Social Research, Faculty of Business and Economics, University of Melbourne, Australia.<br>[3] Centre for Mental Health, Melbourne School of Population and Global Health, University of Melbourne, Australia.
Corresponding author	Dr Owen Bradfield, PhD Candidate, Law and Public Health Unit, Centre for Health Policy, Melbourne School of Population and Global Health, University of Melbourne, 207 Bouverie Street, Carlton, VIC 3053, Australia.<br>Email: owenmb@student.unimelb.edu.au.<br>ORCID ID: 0000-0002-8955-7432
Word count	**3405**

For peer review only

ABSTRACT

Objective: To assess the association between medical negligence claims and doctors' self-rated health and life satisfaction.

Design: Prospective cohort study

Participants: Registered doctors practising in Australia who participated in waves 4 to 11 of the Medicine in Australia: Balancing Employment and Life (MABEL) longitudinal survey between 2011 and 2018.

Primary and secondary outcome measures: Self-rated health and self-rated life satisfaction.

Results: Of the 15,105 doctors in the study, 885 reported being named in a medical negligence claim. Fixed-effects linear regression analysis showed that both self-rated health and self-rated life satisfaction declined for all doctors over the course of the MABEL survey, with no association between wave and being sued. However, being sued was not associated with any additional declines in self-rated health (coef. = -0.01, 95% CI -0.05 to 0.02, p=0.41) or self-rated life satisfaction (coef. = -0.01, 95% CI -0.08 to 0.06, p=0.83) after controlling for a range of job factors. Instead, we found that working conditions and job satisfaction were the strongest predictors of self-rated health and self-rated life satisfaction in sued doctors. In analyses restricted to doctors who were sued, we observed no changes in self-rated health (p=0.99) or self-rated life satisfaction (p=0.59) in the years immediately following a claim.

Conclusions: In contrast to prior overseas cross-sectional survey studies, we show that medical negligence claims do not adversely affect the wellbeing of doctors in Australia, when adjusting for time trends and previously established covariates. This may be because: 1) prior studies failed to adequately address issues of causation and confounding; 2) legal processes governing medical negligence claims in Australia cause less distress compared to those in other jurisdictions. Our findings suggest that the interaction between medical negligence claims and poor doctors' health is more complex than revealed through previous studies.

Abstract word count: 293

For peer review only

Article summary:

Strengths and limitations of our study

- The key strength of this study is that it is the first to examine whether experiencing a medical negligence claim is associated with a change in self-rated health and self-rated life satisfaction over time, when adjusting for common covariates. Prior research has largely relied on cross-sectional surveys that cannot distinguish predictors from consequences of medical negligence claims (that is, whether poor health increases the risk of being sued or whether being sued increases the risk of poor health). Here, the use of seven years of cohort data, and the ability to adjust for demographic, vocational, and psychosocial factors that contribute to poor doctor health, enabled clearer causal inferences to be drawn between medical negligence claims and psychosocial outcomes.
- The main limitations were: 1) Doctors were lost to follow up at the end of each wave, which may have resulted in a selection bias in that doctors with poorer health may have been less likely to remain in successive waves of the survey; 2) Data on exposure and outcome variables were based on self-report; and 3) Only a small proportion of doctors participating in the survey were sued, which might have reduced our capacity to detect a statistically significant difference in self-rated health and life satisfaction between sued doctors and controls.

Acknowledgements: This research used data from the MABEL longitudinal survey of doctors. MABEL was developed by the Melbourne Institute of Applied Economic and Social Research and Monash University, Melbourne, Australia. Funding for MABEL has been provided by the National Health and Medical Research Council (2007 to 2016: 454799 and 1019605); the Australian Government Department of Health and Ageing (2008); Health Workforce Australia (2013); and in 2017 The University of Melbourne, Medibank Better Health Foundation, the NSW Ministry of Health, and the Victorian Department of Health and Human Services. In 2018, funding was provided by the Australian Government Department of Health.

For peer review only - http://bmjopen.bmj.com/site/about/guidelines.xhtml

Contributors: OB and MS developed the initial idea for the study. MB and AS revised the draft critically for important intellectual content. All authors have given final approval for the article to be published.

Funding statement: Dr Owen Bradfield was funded by the Australian Government on a 2020 Melbourne Research Scholarship and is a 2020 Fulbright Future Scholar. A/Prof Marie Bismark was funded by the Australian Government on a National Health and Medical Research Council Investigator Grant (APP1195984). A/Prof Matthew Spittal was funded by the Australian Government on an Australian Research Council Future Fellowship (FT180100075).

Competing interests: None

Provenance and peer review: Not commissioned; externally peer reviewed.

Data sharing statement: De-identified MABEL survey data are available upon application to the Australian Data Archive.

For peer review only

Introduction

Medical negligence litigation allows patients who suffer injury or loss resulting from substandard medical care to seek redress through the legal system.[1] However, the system has been criticised for being slow, expensive, inaccessible,[2] and emotionally distressing for patients and doctors.[3] This is a societal problem,[4] as accumulating research shows that poor doctor wellbeing adversely affects doctors' clinical performance and decision-making, which can directly affect the quality of care that patients receive.[5]

Prior cross-sectional studies in the UK,[6] Ireland,[7] US,[8] and Canada[9] have converged on the conclusion that medical negligence litigation adversely impacts doctors' health. For example, a recent self-report survey of nearly 8000 UK doctors found that doctors with a current or recent claim were approximately twice as likely as doctors without a claim to report suicidal ideation or moderate-severe depression and anxiety. Another study showed that doctors who were sued experienced higher rates of depression, anxiety, post-traumatic stress disorder, and suicidal ideation than doctors who were not.[10] Doctors who were sued identified the litigation process as their most stressful life experience: more so than divorce or the death of a spouse.[11] Even doctors who have not been sued identified the 'threat of litigation' as their most serious work-related stressor[12] and this was associated with doctors leaving the profession.[13]

The principal limitation of this prior body of research is it almost entirely relies on cross-sectional surveys[14] that cannot easily disentangle the temporal relationship between the causes and consequences of medical negligence claims. This limits the ability to infer a causal association and means, for instance, that it is not possible to reliably untangle whether being sued increases the risk of poor health or whether poor health increases the risk of being sued, or both. It also limits the extent to which known risk factors for poor doctor health, including high job demands, low job control, and low work-life balance can be considered as confounders or alternative explanations of any exposure-outcome association. These issues could be resolved using prospective cohort data because individuals can be followed over time, allowing observation of outcomes before and after a claim. It would also allow the impact of confounders on the exposure-outcome association to be observed.

In this study, we used the Medicine in Australia: Balancing Employment and Life (MABEL) cohort to assess the relationship between self-reported medical negligence claims and self-rated

For peer review only - http://bmjopen.bmj.com/site/about/guidelines.xhtml

health and self-rated life satisfaction. We used a fixed-effects regression approach to control for stable individual factors that are not observed in the data as well as time-varying observed factors. Based on prior studies, we hypothesised that, compared to doctors who were not sued, doctors who were sued would experience poorer psychological outcomes when controlling for confounding factors (measures of job quality). We further hypothesised that among doctors who were sued, there would be a decline in psychological outcomes in the year of a claim and in the year or two following a claim when compared to their outcomes prior to being sued. Our primary outcome measure was self-rated health, and our secondary outcome measure was self-rated life satisfaction.

Methods

Data source

MABEL is a longitudinal panel survey of doctors' working conditions, job satisfaction, work-life balance, health, and life satisfaction. It comprised 11 annual waves that were collected between 2008 and 2018. The initial cohort was drawn from a national directory of 59,620 practicing doctors in Australia, with new medical graduates and newly registered overseas trained doctors invited to participate in subsequent waves. These new recruits replaced doctors lost to follow-up and maintained the cross-sectional representativeness of the cohort. MABEL excluded doctors who had retired, were on leave, or working overseas. MABEL was developed by researchers at the Melbourne Institute of Applied Economic and Social Research and Monash University, Melbourne, Australia. Copies of the survey instruments are publicly available[15] and a detailed description of the MABEL protocol and cohort has been published elsewhere.[16]

Sample selection

We restricted our analysis to survey responses from waves four to 11 (2011 to 2018) because earlier waves did not include questions about medical negligence claims. We excluded doctors-in-training and hospital non-specialists because they are usually hospital employees. In Australia, hospitals are vicariously liable for the negligence of doctors within their employ.[17] This means that the hospital, rather than its employed doctors, would defend medical any negligence litigation.

Variables of interest

For peer review only - http://bmjopen.bmj.com/site/about/guidelines.xhtml

Our primary outcome was self-rated health. The survey question was "In general, would you say your health is: excellent, very good, good, fair, poor". We recoded this variable so that higher scores indicated better health. This tool is a strong predictor of mortality[18] and has been used in other large health research surveys.[19] Our secondary outcome was self-rated life satisfaction. This was measured using a 10-point scale asking respondents to self-rate their life satisfaction from 1 ("completely dissatisfied") to 10 ("completely satisfied"). Higher scores are indicative of greater satisfaction. Surveys of life satisfaction have been shown to be stable and sensitive to changing life circumstances.[20]

Our key exposure variable was being a defendant in a medical negligence claim. This was constructed by combining responses to two questions: "In the preceding 12 months have you been named in a medical negligence claim?" (yes or no); and "how long ago did it happen?" (≤3 months, 4-6 months, 7-9 months, 10-12 months ago). Responses were coded 0 for all waves prior to being sued and 1 from the wave the respondent reported being sued onwards. Doctors who were not sued during the study period were coded 0 throughout.

High job demands, low job control, poor social supports, and work-life imbalance have all been previously associated with higher odds of poorer self-rated health.[21] Therefore, to adjust for the potential confounding effect of these variables, as well as age and hours worked per week, we included indicators of each of these in our models. High job demands were measured using four items scored 0 to 4 (strongly disagree to strongly agree). These were "It is difficult to take time off when I want to", "My patients have unrealistic expectations about how I can help them", "Running my practice is stressful most of the time" and "The majority of my patients have complex health and social problems". Low job control was measured using five items: "Freedom to choose your own method of working?" (scored from 0 "very dissatisfied" to 4 "very satisfied", reverse coded); "Amount of variety in your work?" (scored from 0 "very dissatisfied" to 4 "very satisfied", reverse coded); "Amount of responsibility you are given" (scored from 0 "very dissatisfied" to 4 "very satisfied", reverse coded); "The hours I work are predictable" (scored from 0 "strongly disagree" to 4 "strongly agree"); and "I am restricted in my employment and/or the time and hours I work due to lack of available childcare" (scored from 0 "strongly disagree" to 4 "strongly agree"). Poor social supports were measured using three items scored 0 to 4 (strongly disagree to strongly agree). These were: "I have a poor support network of other doctors like me"; "I don't have many friends or family members in

For peer review only

my current work location"; and "It is easy to pursue my hobbies and leisure interests in my current work location" (reverse coded).

Work-life balance was measured using four items: "The balance between my personal and professional commitments is about right" (scored from 0 "strongly disagree" to 4 "strongly agree"), "My hours of work" (scored from 0 "very dissatisfied" to 4 "very satisfied"), "I can take time off at short notice, for example if one of my children is ill or for a home emergency" (scored from 0 "strongly disagree" to 4 "strongly agree") and "My colleagues understand the need for work-life balance" (scored from 0 "strongly disagree" to 4 "strongly agree").

High job demands, low job control, poor social support and work-life balance were all converted to z scores with means of 0 and standard deviations of 1. Age at each wave was coded into 5-year bands (<35, 35-39, 40-44, 45-49, 50-54, 55-59, 60-64, 65-69, ≥70 years). Working hours was coded into three categories based on the Australian Bureau of Statistics' definition of a standard full-time working week.[22] These were <35 hours per week, 35 to 45 hours per weeks and >45 hours per week.

We also had information on the following time-invariant variables that we set to their baseline values: sex (male, female); speciality (general practitioners, adult medicine physicians, surgeons, paediatricians, anaesthetists, pathologists and radiologists, emergency physicians, obstetricians and gynaecologists, ophthalmologists, psychiatrists, other); dependent children (collapsed into "none" or "one or more"); currently living with partner or spouse (yes or no); and geographical location (collapsed into three categories based on the five-category Australian Standard Geographical Classification[23]).

Statistical analyses

We first described the characteristics of the cohort at baseline using counts and percentages. We then performed fixed-effects linear regression analyses to examine changes in self-rated health and self-rated life satisfaction. Fixed effects models are useful in the context of cohort data because they estimate the average within-person change in the outcome according to time-varying covariates entered into the model. Our key predictor was the binary variable representing the wave a doctor reported being sued onwards. We adjusted for wave, hours worked, high job demands, low job control, poor social supports, work-life balance, and age.

For peer review only

We also performed post hoc tests to determine if there was an interaction (effect modification) between being sued and wave for each outcome. Finally, to explore a possible temporal relationship between being sued and the outcomes, we undertook an analysis only among those doctors who reported being sued. Our key predictor was a variable that distinguished between the years prior to being sued, the year they were sued, 1 year after being sued, two years after being sued, and so on. Thus, we used fixed effects methods to determine if self-rated health and satisfaction with life changed after doctors were sued, and if so, how long any change persisted for. Our models included the same set of covariates as the main models. All analyses were conducted using Stata 16.1 (StataCorp, College Station, TX, USA).

Ethics

The MABEL survey was approved by The University of Melbourne Faculty of Business and Economics Human Ethics Advisory Group (Ref. 0709559) and the Monash University Standing Committee on Ethics in Research Involving Humans (Ref. CF07/1102 -2007000291). Our study was approved by the Melbourne School of Population and Global Health Human Ethics Advisory Group (Ref. 1956096). The Melbourne Institute: Applied Economic and Social Research granted access to de-identified MABEL survey responses. Participant consent for publication was not required.

Patient and public involvement

It was not possible to involve patients or the public in the design, conduct, reporting, or dissemination plans of our research. Results will be made available to MABEL participants at https://melbourneinstitute.unimelb.edu.au/mabel/results-and-publications/journal-articles.

Results

Demographic details of survey participants

Between 2011 and 2018, 15,105 doctors were available for analysis. 885 (5.90%) reported being sued at least once. The characteristics of doctors included for analysis are shown in Table 1. They were predominately male (56%), general practitioners (53%), working more than 45 hours per week (28%), and practicing in metropolitan areas (70%). The majority were living with their spouse or partner (85%) and had at least one dependent child (61%). The baseline mean score for self-rated health was 3.07 (SD = 0.92, range 0 to 4), where the maximum score

For peer review only - http://bmjopen.bmj.com/site/about/guidelines.xhtml

represented excellent health. The mean self-rated life satisfaction score was 7.42 (SD = 1.62, range 1 to 10) where the maximum score represented complete satisfaction. Doctors were followed for between 1 and 8 waves, with a mean of 3.8 waves. One third of doctors (n = 5,847) completed 5 or more of the 8 waves of data collection. 18% of doctors reported being sued in the first wave (2011) and 7% reported being sued in the last wave (2018). The remaining 75% reported being sued in the intervening waves with the proportion sued for the first time declining in each wave.

Association between being sued and self-rated health and life satisfaction

Multivariate fixed-effects linear regression analysis indicated that mean self-rated health declined each wave (coef. = -0.04, 95% CI -0.05 to -0.04, $p < 0.001$) (Table 2). There was no evidence that being sued was associated with any additional declines in self-rated health (coef. = -0.02, 95% CI -0.06 to 0.02, $p = 0.39$). High job demands (coef. = -0.02 per 1 standard deviation [SD] increase in scores, 95% CI -0.03 to -0.01, $p < 0.001$), low job control (coef. = -0.04 per SD increase, 95% CI -0.05 to -0.03, $p<0.001$), and poor social supports (coef. = -0.03 per SD increase, 95% CI -0.04 to -0.02, $p<0.001$) were all associated with lower self-rated health. Achieving work-life balance (coef. = 0.04 per SD increase, 95% CI 0.03 to 0.05, $p < 0.001$) was associated with higher self-rated health. There was no evidence that hours worked per week ($p = 0.21$) or age ($p = 0.14$) were associated with self-rated health. In a post hoc test there was no evidence of an interaction between wave and being sued ($p = 0.13$).

A similar set of findings emerged for self-rated life satisfaction. Mean life satisfaction declined during each wave of data collection (coef = -0.06, 95% CI -0.07 to -0.05, $p<0.001$) and being sued was not associated with any additional decline (coef. = -0.01, 95% CI -0.08 to 0.07, $p = 0.91$). Results for all other predictors were similar, as detailed in Table 2, and in a post hoc test there was no evidence of an interaction between wave and being sued ($p = 0.42$).

Temporal association between being sued and self-rated health and life satisfaction

Among doctors who had been sued, we found no evidence that self-rated health or self-rated life satisfaction changed in the years after a claim was made (Table 3). Compared to a sued doctors' self-rated health in the years prior to a claim, there was no evidence that their health changed in the year of a claim (coef. = -0.01. 95% CI -0.07 to 0.06), the year after the claim (coef. = -0.01, 95% CI -0.09 to 0.07) or any of the other subsequent years. The same pattern of results was repeated for life-satisfaction.

For peer review only - http://bmjopen.bmj.com/site/about/guidelines.xhtml

Discussion

Doctors' health has recently been described as a "global health-care predicament", with emerging evidence that poor doctor wellbeing adversely affects healthcare quality and safety.[24] Doctors who are unwell take more time off work, leading to workforce understaffing, increased staff turnover, and increased healthcare expenditure.[25] Similarly, studies have linked poor doctor health with suboptimal patient care and a doubling of the risk of medical errors.[26] Factors contributing to poor doctor health include: professional stressors (long working hours, shift work, workplace violence); a blame culture in medicine; fear and stigma of discussing health concerns with colleagues; and easy access to medications that leads to self-prescribing.[27] There is also mounting research suggesting that medico-legal claims and complaints may contribute to poor doctor health.[28]

Providing good patient care is central to doctors' professional identities. Doctors are notoriously self-critical and interpret allegations made against them during litigation as an assault on their professional competence and integrity,[29] which can lead to re-traumatisation[30] and vocational disenchantment.[31] Litigation can also inflict financial and reputational damage,[32] while fear of future litigation can lead to defensive behaviours such as over-investigation or avoidance of high-risk patients and procedures.[33] These effects may be compounded by legal advice that discourages doctors from speaking about their litigation experience with colleagues and peers for fear of compromising their claim or breaching confidential settlement terms.[34]

However, in contrast to previous research, our study did not find an association between medical negligence claims and self-rated health and self-rated life satisfaction. There are possible several explanations for this. First, prior studies adopted a cross-sectional design, which means that causation and the impact of time or confounders could not be verified. Second, prior studies often examined the association between doctor health and various types of medico-legal claims, such as complaints or regulatory investigations, rather than medical negligence litigation specifically. Complaints and regulatory investigations may affect doctors more severely, or in different ways, than litigation. Third, the legal processes and frameworks governing medical negligence claims differ between jurisdictions. Those processes in Australia may cause less distress than processes overseas.

For peer review only - http://bmjopen.bmj.com/site/about/guidelines.xhtml

In Australia, tort law reforms enacted 20 years ago aimed at curtailing medical negligence litigation may have positively impacted the litigation experience for doctors in Australia.[35] Following widespread concerns that the volume and cost of medical litigation was making medical indemnity insurance unaffordable and unavailable for many doctors,[36] reforms were introduced that included: i) shortening time-limits within which proceedings may be initiated; ii) limiting claims to "significant" injuries; iii) capping compensation payments; and iv) mandating mediation.[37] It is also compulsory for Australian doctors to have professional indemnity insurance.[38] As a result of these reforms, there are fewer medical negligence claims against doctors. In our study, the proportion of doctors who reported being sued for the first time declined year on year. Of those medical negligence claims that are commenced, the overwhelming majority settle out of court on confidential terms. This may mean that Australian medical negligence claims are rarely subject to media scrutiny and are less likely to inflict financial or reputational damage on doctors. In addition, medical defence insurers and legal practitioners play a crucial role in supporting and educating sued doctors about the personal and professional impacts of legal processes.[39] Our results may also suggest that sued doctors in Australia are better supported professionally and personally, compared to overseas.

Nevertheless, despite the absence of a correlation between medical negligence claims and poor doctor health, our findings add weight to growing calls to improve doctors' health. Self-rated health and self-rated life-satisfaction declined on average throughout the duration of the study. Growing demands on doctors, associated with higher patient expectations, and increased administrative and regulatory requirements, may have contributed to this finding. We found that high job demands, low job control, poor social support, and a work-life balance were all associated with self-rated health and life satisfaction. This is consistent with previous findings that showed a correlation between poor psychosocial working conditions and self-rated health in doctors.[40] It is likely that workplace factors have significantly contributed to declining doctor health over the seven years studied. This reinforces the pressing need for ongoing efforts to support doctors' health and wellbeing, particularly during the pandemic. Doctors are often ashamed to disclose to peers that they are unwell for fear of being judged. Being unable to share their experiences may exacerbate feelings of isolation. As a group, unwell doctors are often silent and invisible with few available avenues of peer support. This needs to change, as prior research shows that doctors enjoy better psychological wellbeing when supported by family, colleagues, or employers.[41]

For peer review only - http://bmjopen.bmj.com/site/about/guidelines.xhtml

Our study had several limitations. First, a number of doctors were lost to follow up at the end of each wave, which may have resulted in a selection bias in that doctors with poorer health may have been less likely to remain in successive waves of the survey. Second, data in relation to exposure to a medical negligence claim and the primary and secondary health and life satisfaction outcomes were self-reported. Third, as only a small proportion of doctors participating in the survey were sued, our study may have lacked sufficient power to detect a statistically significant difference in self-rated health and life satisfaction between sued doctors and controls. Despite these limitations, the key strengths of this study were: its large sample size; the prospective cohort study design that enabled us to draw stronger causal inferences than previous studies; the assessment of a wide range of demographic, vocational, and psychosocial covariables; and controlling for time-invariant bias within and between persons.

While there are reports of doctors who have died by suicide in the context of medical regulatory investigations,[42] our large longitudinal analysis of doctors in Australia found no association between medical negligence claims and poor doctors' health. This may be because medical negligence claims have less impact on doctors compared to regulatory complaints or investigations. It may also be because any adverse impact of claims on doctors' health is short-lived and does not translate into lower self-rated health or life satisfaction scores 12 months later. Instead, we found a significant association between workplace factors and doctors' health, suggesting that workplace health and safety reforms, rather than further tort law reforms, ought to be a priority for continued work in improving the health and wellbeing of [illegible]

For peer review only - http://bmjopen.bmj.com/site/about/guidelines.xhtml

References:

[1] Vincent C, Young M, Phillips A. Why do people sue doctors? A study of patients and relatives taking legal action. *Lancet*. 1994;343(8913):1609-13. doi: 10.1016/s0140-6736(94)93062-7. PMID: 7911925.

[2] Bielen S, Grajzl P, Marneffe W. The resolution process and the timing of settlement of medical malpractice claims. *Health Econ Policy Law*. 2020;15(4):509-529. doi: 10.1017/S1744133119000185. Epub 2019 Apr 17. PMID: 30994084.

[3] Nash L, Curtis B, Walton M, Willcock S, Tennant C. The response of doctors to a formal complaint. *Australas Psychiatry*. 2006 Sep;14(3):246-50. doi: 10.1080/j.1440-1665.2006.02295.x. PMID: 16923032.

[4] Keet M, Heavin H, Sparrow S. Anticipating and managing the psychological cost of civil litigation. *Windsor Yearbook of Access to Justice/Recueil annuel de Windsor d'accès à la justice*. 2017;34(2):73-98, at 85.

[5] Wallace JE, Lemaire JB, Ghali WA. Physician wellness: a missing quality indicator. *Lancet*. 2009 Nov 14;374(9702):1714-21. doi: 10.1016/S0140-6736(09)61424-0. PMID: 19914516.

[6] Bourne T, Wynants L, Peters M, Van Audenhove C, Timmerman D, Van Calster B, Jalmbrant M. The impact of complaints procedures on the welfare, health and clinical practise of 7926 doctors in the UK: a cross-sectional survey. *BMJ Open*. 2015 Jan 15;5(1):e006687. doi: 10.1136/bmjopen-2014-006687. PMID: 25592686; PMCID: PMC4316558.

[7] Tumelty, M. Exploring the emotional burdens and impact of medical negligence litigation on the plaintiff and medical practitioner: Insights from Ireland. *Legal Studies,* 2021; 1-24. doi:10.1017/lst.2021.20.

[8] Balch CM, Oreskovich MR, Dyrbye LN, Colaiano JM, Satele DV, Sloan JA, Shanafelt TD. Personal consequences of malpractice lawsuits on American surgeons. *J Am Coll Surg*. 2011 Nov;213(5):657-67. doi: 10.1016/j.jamcollsurg.2011.08.005. Epub 2011 Sep 3. PMID: 21890381.

[9] Cook WR, Neff C. Attitudes of physicians in northern Ontario to medical malpractice litigation. *Can Fam Physician*. 1994; 40:689-98. PMID: 8199521; PMCID: PMC2380112.

[10] Jain A, Ogden J. General practitioners' experiences of patients' complaints: qualitative study. *BMJ*. 1999 Jun 12;318(7198):1596-9. doi: 10.1136/bmj.318.7198.1596. PMID: 10364121; PMCID: PMC28140.

[11] Charles SC, Warnecke RB, Nelson A, Pyskoty CE. Appraisal of the event as a factor in coping with malpractice litigation. *Behav Med*. 1988 Winter;14(4):148-55. doi: 10.1080/08964289.1988.9935139. PMID: 3256367.

[12] Schattner PL, Coman GJ. The stress of metropolitan general practice. *Med J Aust*. 1998 Aug 3;169(3):133-7. doi: 10.5694/j.1326-5377.1998.tb116013.x. PMID: 9734508.

[13] Charles SC, Wilbert JR, Franke KJ. Sued and nonsued physicians' self-reported reactions to malpractice litigation. *Am J Psychiatry*. 1985 Apr;142(4):437-40. doi: 10.1176/ajp.142.4.437. PMID: 3976916.

[14] Nash LM, Kelly PJ, Daly MG, Walter G, van Ekert EH, Walton M, Willcock SM, Tennant CC. Australian doctors' involvement in medicolegal matters: a cross-sectional self-report study. *Med J Aust*. 2009 Oct 19;191(8):436-40. doi: 10.5694/j.1326-5377.2009.tb02879.x. PMID: 19835537.

[15] Medicine in Australia: Balancing Employment and Life. University of Melbourne. Available at: https://melbourneinstitute.unimelb.edu.au/mabel (accessed 29 June 2021).

[16] Joyce CM, Scott A, Jeon S-H, Humphreys J, Kalb G, Witt J, Leahy A. The "Medicine in Australia: Balancing Employment and Life (MABEL)" longitudinal survey--protocol and baseline data for a prospective cohort study

For peer review only - http://bmjopen.bmj.com/site/about/guidelines.xhtml

of Australian doctors' workforce participation. *BMC Health Serv Res* 2010; 10(1): 1–10. doi: 10.1186/1472-6963-10-50. PMID: 20181288; PMCID: PMC2837653.

[17] White B, McDonald FJ, Willmott L. *Health law in Australia*. Lawbook Company; 2014: 375.

[18] Idler EL, Benyamini Y. Self-rated health and mortality: a review of twenty-seven community studies. J Health Soc Behav. 1997 Mar;38(1):21-37. PMID: 9097506.

[19] Subramanian SV, Huijts T, Avendano M. Self-reported health assessments in the 2002 World Health Survey: how do they correlate with education?. *Bulletin of the World Health Organization*. 2010; 88:131-8.

[20] Siahpush M, Spittal M, Singh GK. Happiness and life satisfaction prospectively predict self-rated health, physical health, and the presence of limiting, long-term health conditions. *Am J Health Promot*. 2008;23(1):18-26. doi: 10.4278/ajhp.061023137. PMID: 18785370.

[21] Milner A, Witt K, Spittal MJ, Bismark M, Graham M, LaMontagne AD. The relationship between working conditions and self-rated health among medical doctors: evidence from seven waves of the Medicine In Australia Balancing Employment and Life (Mabel) survey. *BMC Health Serv Res*. 2017 Aug 29;17(1):609. doi: 10.1186/s12913-017-2554-z. PMID: 28851354; PMCID: PMC5576303.

[22] ABS. Underemployed Workers, Australia, September 2013. Cat. No. 6265.0. Canberra: Australian Bureau of Statistics; 2013.

[23] Australian Bureau of Statistics (2006) *Australian Standard Geographical Classification (ASGC)*. Australian Bureau of Statistics, Canberra. Available at: https://www.abs.gov.au/websitedbs/d3310114.nsf/home/australian+standard+geographical+classification+(asgc). Accessed 2 July 2021.

[24] The Lancet. Physician burnout: the need to rehumanise health systems. *Lancet*. 2019 Nov 2;394(10209):1591. doi: 10.1016/S0140-6736(19)32669-8. PMID: 31690430.

[25] Carayon P, Cassel C, Dzau VJ. Improving the System to Support Clinician Well-being and Provide Better Patient Care. *JAMA*. 2019 Dec 10;322(22):2165-2166. doi: 10.1001/jama.2019.17406. PMID: 31644783.

[26] Fahrenkopf AM, Sectish TC, Barger LK, et al. Rates of medication errors among depressed and burnt out residents: prospective cohort study. *BMJ* 2008; 336(7642): 488-91. doi: 10.1136/bmj.39469.763218.BE. Epub 2008 Feb 7. PMID: 18258931; PMCID: PMC2258399.

[27] National Academies of Sciences, Engineering, and Medicine; National Academy of Medicine; Committee on Systems Approaches to Improve Patient Care by Supporting Clinician Well-Being. Taking Action Against Clinician Burnout: A Systems Approach to Professional Well-Being. Washington (DC): National Academies Press (US); 2019 Oct 23. PMID: 31940160.

[28] Browne P, Haysom G. Supportive networks, healthier doctors and 'just culture': Managing the effects of medico-legal complaints on doctors. *Aust J Gen Pract*. 2019 Jan-Feb;48(1-2):9-12. doi: 10.31128/AJGP-09-18-4713. PMID: 31256451.

[29] American College of Obstetricians and Gynecologists. ACOG Committee opinion no. 551: coping with the stress of medical professional liability litigation. *Obstet Gynecol*. 2013; 121(1): 220-2. doi: 10.1097/01.aog.0000425665.64635.1c. PMID: 23262965.

[30] Scott SD, Hirschinger LE, Cox KR, McCoig M, Brandt J, Hall LW. The natural history of recovery for the healthcare provider "second victim" after adverse patient events. *Qual Saf Health Care*. 2009;18(5):325-30. doi: 10.1136/qshc.2009.032870. PMID: 19812092.

For peer review only - http://bmjopen.bmj.com/site/about/guidelines.xhtml

[31] Debono B, Hamel O, Guillain A, Durand A, Rué M, Sabatier P, Lonjon G, Dran G; French Society of Private Neurosurgeons (SFNCL). Impact of malpractice liability among spine surgeons: A national survey of French private neurosurgeons. *Neurochirurgie*. 2020 Aug;66(4):219-224. doi: 10.1016/j.neuchi.2020.05.003. Epub 2020 Jun 12. PMID: 32540341.

[32] Nash L, Tennant C, Walton M. The psychological impact of complaints and negligence suits on doctors. *Australas Psychiatry*. 2004 Sep;12(3):278-81; discussion 282. doi: 10.1080/j.1039-8562.2004.02079.x. PMID: 15715791.

[33] Reisch LM, Carney PA, Oster NV, Weaver DL, Nelson HD, Frederick PD, Elmore JG. Medical malpractice concerns and defensive medicine: a nationwide survey of breast pathologists. *Am J Clin Pathol*. 2015 Dec;144(6):916-22. doi: 10.1309/AJCP80LYIMOOUJIF. PMID: 26572999; PMCID: PMC6436915.

[34] Eisenberg RL. Reactions of physicians sued for malpractice. In: Radiology and the Law 2004 (pp. 74-77). Springer, New York, NY.

[35] Panel of Eminent Persons to Review the Law of Negligence (the Ipp Committee), *Review of the Law of Negligence, Final Report.* Canberra, October 2002. Available at: https://treasury.gov.au/sites/default/files/2019-03/R2002-001_Law_Neg_Final.pdf (accessed 16 July 2021)

[36] Luntz, H. (2002). Reform of the Law of Negligence: Wrong Questions - Wrong Answers. *UNSW Law Journal*, 25(3), 836–841. doi:10.3316/informit.119529917834518.

[37] Skene L. Law and medical practice: rights, duties, claims and defences. 3rd ed. Australia: LexisNexisButterworths, 2008.

[38] *Health Practitioner Regulation National Law Act 2009* (Qld), section 129.

[39] Haysom G. The impact of complaints on doctors. *Aust Fam Physician*. 2016;45(4):242-4. PMID: 27052144.

[40] Elovainio M, Salo P, Jokela M, Heponiemi T, Linna A, Virtanen M, Oksanen T, Kivimäki M, Vahtera J. Psychosocial factors and well-being among Finnish GPs and specialists: a 10-year follow-up. *Occup Environ Med*. 2013 Apr;70(4):246-51. doi: 10.1136/oemed-2012-100996. Epub 2013 Jan 15. PMID: 23322916.

[41] Bourne T, De Cock B, Wynants L, Peters M, Van Audenhove C, Timmerman D, Van Calster B, Jalmbrant M. Doctors' perception of support and the processes involved in complaints investigations and how these relate to welfare and defensive practice: a cross-sectional survey of the UK physicians. *BMJ Open*. 2017 Nov 21;7(11):e017856. doi: 10.1136/bmjopen-2017-017856. PMID: 29162574; PMCID: PMC5719304.

[42] Horsfall S. *Doctors who commit suicide while under GMC fitness to practice investigation* (GMC Report, 14 December 2014) < https://www.gmc-uk.org/-/media/documents/Internal_review_into_suicide_in_FTP_processes.pdf_59088696.pdf> (accessed 8 November 2021).

For peer review only - http://bmjopen.bmj.com/site/about/guidelines.xhtml

Table 1: Characteristics of doctors at baseline (n = 15,105)

Characteristic	n	%
Sex		
Female	6,718	44.5
Male	8,334	55.2
Missing	53	0.4
Age group		
<35	2,387	15.8
35-39	2,376	15.7
40-44	2,117	14.0
45-49	1,873	12.4
50-54	1,832	12.1
55-59	1,585	10.5
60-64	1,105	7.3
65-69	702	4.6
≥70	634	4.2
Missing	494	3.3
Speciality		
General practitioner	7,539	49.9
Adult medicine	1,760	11.7
Surgery	686	4.5
Paediatrics	427	2.8
Anaesthesia	1,007	6.7
Pathology and radiology	437	2.9
Emergency	403	2.7
Obstetrics and gynaecology	393	2.6
Ophthalmology	181	1.2
Psychiatry	634	4.2
Dermatology	68	0.5
Other	612	4.1
Missing	958	6.3
Work location		
Metropolitan	10,097	66.8
Regional/Rural	2,775	18.4
Remote	1,515	10.0
Missing	718	4.8
Hours worked per week		
<35	5,081	33.6
35-45	5,149	34.1
>45	4,182	27.7
Missing	693	4.6
Dependent children		
None	5,196	34.4
One or more	8,318	55.1
Missing	1,591	10.5
Living with a spouse or partner		
Yes	11,623	76.9
No	2,036	13.5
Missing	1,446	9.6

Table 2: Fixed effects regression predicting self-rated health and satisfaction with life

	**Self-rated health (51,099 observations among 13,841 doctors)**		**Satisfaction with life (51,119 observations among 13,821 doctors)**	
	Coefficient (95% CI)	p value	Coefficient (95% CI)	p value
**Medical negligence claim**		0.40		0.91
No	Ref.		Ref.	
Yes	-0.02 (-0.06 to 0.02)		-0.01 (-0.08 to 0.07)	
**Wave** (per 1 wave increase)	-0.04 (-0.05 to -0.04)	<0.001	-0.06 (-0.07 to -0.05)	<0.001
**Hour of work per week**		0.22		0.034
<35 hours	Ref.		Ref.	
35-45 hours	0.02 (-0.01 to 0.03)		0.03 (-0.01 to 0.06)	
>45 hours	0.02 (-0.01 to 0.04)		-0.01 (-0.06 to 0.03)	
**High job demands** (per 1 SD increase)	-0.02 (-0.03 to -0.01)	<0.001	-0.11 (-0.12 to -0.09)	<0.001
**Low job control** (per 1 SD increase)	-0.04 (-0.05 to -0.03)	<0.001	-0.25 (-0.27 to -0.24)	<0.001
**Poor social supports** (per 1 SD increase)	-0.03 (-0.04 to -0.02)	<0.001	-0.15 (-0.16 to -0.13)	<0.001
**Work life balance** (per 1 SD increase)	0.04 (0.03 to 0.05)	<0.001	0.21 (0.19 to 0.22)	<0.001
**Age**		0.14		<0.001
≤35 years	Ref.		Ref.	
35-39 years	-0.03 (-0.07 to 0.01)		0.01 (-0.07 to 0.09)	
40-44 years	-0.05 (-0.10 to 0.01)		-0.07 (-0.17 to 0.03)	
45-49 years	-0.04 (-0.10 to 0.03)		-0.18 (-0.30 to -0.06)	
50-54 years	-0.03 (-0.10 to 0.05)		-0.23 (-0.37 to -0.09)	
55-59 years	-0.03 (-0.12 to 0.05)		-0.21 (-0.37 to -0.05)	
60-64 years	-0.01 (-0.11 to 0.09)		-0.17 (-0.35 to 0.01)	
65-69 years	0.03 (-0.08 to 0.14)		-0.06 (-0.26 to 0.15)	
≥70 years	0.04 (-0.09 to 0.17)		0.01 (-0.24 to 0.25)	

For peer review only

For peer review only

Table 3: Effect of time since claim on self-rated health or life satisfaction in doctors who were sued

	Self-rated health (4,615 observations among 882 doctors)		Self-rated life satisfaction (4,713 observations among 885 doctors)	
	**Coefficient (95% CI)**	**p value**	**Coefficient (95% CI)**	**p value**
**Time since claim**		0.99		0.82
Years prior to a claim	Ref.		Ref.	
Year of claim	-0.01 (-0.07 to 0.06)		-0.07 (-0.19 to 0.05)	
One year after claim	-0.01 (-0.09 to 0.07)		-0.03 (-0.18 to 0.12)	
Two years after a claim	0.01 (-0.08 to 0.10)		-0.07 (-0.24 to 0.11)	
Three years after a claim	-0.01 (-0.11 to 0.10)		-0.11 (-0.31 to 0.10)	
Four or more years after a claim	-0.02 (-0.15 to 0.11)		-0.08 (-0.33 to 0.17)	
**Wave** (per 1 wave increase)	-0.05 (-0.07 to -0.03)	<0.001	-0.05 (-0.08 to -0.02)	0.006
**Hour of work per week**		0.11		0.009
<35 hours	Ref.		Ref.	
35-45 hours	0.02 (-0.04 to 0.08)		0.12 (0.01 to 0.24)	
>45 hours	0.07 (-0.01 to 0.14)		0.21 (0.08 to 0.35)	
**High job demands** (per 1 SD increase)	-0.01 (-0.04 to 0.02)	0.68	-0.08 (-0.14 to -0.02)	0.006
**Low job control** (per 1 SD increase)	-0.05 (-0.07 to -0.02)	0.001	-0.34 (-0.39 to -0.29)	<0.001
**Poor social supports** (per 1 SD increase)	-0.04 (-0.07 to -0.02)	0.001	-0.22 (-0.27 to -0.17)	<0.001
**Work life balance** (per 1 SD increase)	0.04 (0.01 to 0.07)	0.003	0.24 (0.19 to 0.29)	<0.001
**Age**		0.041		0.012
≤35 years	Ref.		Ref.	
35-39 years	-0.21 (-0.38 to 0.04)		0.25 (-0.58 to 0.07)	
40-44 years	-0.23 (-0.44 to 0.02)		-0.49 (-0.89 to -0.09)	
45-49 years	-0.10 (-0.34 to 0.15)		-0.52 (-0.99 to -0.05)	
50-54 years	-0.07 (-0.34 to 0.21)		-0.54 (-1.07 to -0.01)	
55-59 years	-0.03 (-0.34 to 0.28)		-0.43 (-1.02 to 0.17)	
60-64 years	0.05 (-0.30 to 0.39)		-0.39 (-1.05 to 0.27)	
65-69 years	0.07 (-0.31 to 0.45)		-0.07 (-0.81 to 0.66)	
≥70 years	0.08 (-0.35 to 0.51)		-0.01 (-0.84 to 0.82)	

STROBE Statement—Checklist of items that should be included in reports of ***cohort studies***

	Item No	Recommendation	Page No
**Title and abstract**	1	(*a*) Indicate the study's design with a commonly used term in the title or the abstract	1
		(*b*) Provide in the abstract an informative and balanced summary of what was done and what was found	1
**Introduction**			
Background/rationale	2	Explain the scientific background and rationale for the investigation being reported	2
Objectives	3	State specific objectives, including any prespecified hypotheses	2
**Methods**			
Study design	4	Present key elements of study design early in the paper	3
Setting	5	Describe the setting, locations, and relevant dates, including periods of recruitment, exposure, follow-up, and data collection	3-6
Participants	6	(*a*) Give the eligibility criteria, and the sources and methods of selection of participants. Describe methods of follow-up	3
		(*b*) For matched studies, give matching criteria and number of exposed and unexposed	n/a
Variables	7	Clearly define all outcomes, exposures, predictors, potential confounders, and effect modifiers. Give diagnostic criteria, if applicable	4-5
Data sources/ measurement	8*	For each variable of interest, give sources of data and details of methods of assessment (measurement). Describe comparability of assessment methods if there is more than one group	4-5
Bias	9	Describe any efforts to address potential sources of bias	n/a
Study size	10	Explain how the study size was arrived at	6
Quantitative variables	11	Explain how quantitative variables were handled in the analyses. If applicable, describe which groupings were chosen and why	3-6
Statistical methods	12	(*a*) Describe all statistical methods, including those used to control for confounding	5
		(*b*) Describe any methods used to examine subgroups and interactions	
		(*c*) Explain how missing data were addressed	
		(*d*) If applicable, explain how loss to follow-up was addressed	
		(*e*) Describe any sensitivity analyses	
**Results**			
Participants	13*	(a) Report numbers of individuals at each stage of study—eg numbers potentially eligible, examined for eligibility, confirmed eligible, included in the study, completing follow-up, and analysed	5-6
		(b) Give reasons for non-participation at each stage	
		(c) Consider use of a flow diagram	
Descriptive data	14*	(a) Give characteristics of study participants (eg demographic, clinical, social) and information on exposures and potential confounders	6-7
		(b) Indicate number of participants with missing data for each variable of interest	6-7
		(c) Summarise follow-up time (eg, average and total amount)	6-7
Outcome data	15*	Report numbers of outcome events or summary measures over time	7-8

For peer review only

Main results	16	(*a*) Give unadjusted estimates and, if applicable, confounder-adjusted estimates and their precision (eg, 95% confidence interval). Make clear which confounders were adjusted for and why they were included<br>(*b*) Report category boundaries when continuous variables were categorized<br>(*c*) If relevant, consider translating estimates of relative risk into absolute risk for a meaningful time period	8
Other analyses	17	Report other analyses done—eg analyses of subgroups and interactions, and sensitivity analyses	n/a
**Discussion**			
Key results	18	Summarise key results with reference to study objectives	8-10
Limitations	19	Discuss limitations of the study, taking into account sources of potential bias or imprecision. Discuss both direction and magnitude of any potential bias	10
Interpretation	20	Give a cautious overall interpretation of results considering objectives, limitations, multiplicity of analyses, results from similar studies, and other relevant evidence	8-10
Generalisability	21	Discuss the generalisability (external validity) of the study results	8-10
**Other information**			
Funding	22	Give the source of funding and the role of the funders for the present study and, if applicable, for the original study on which the present article is based	See title page

*Give information separately for exposed and unexposed groups.

Note: An Explanation and Elaboration article discusses each checklist item and gives methodological background and published examples of transparent reporting. The STROBE checklist is best used in conjunction with this article (freely available on the Web sites of PLoS Medicine at http://www.plosmedicine.org/, Annals of Internal Medicine at http://www.annals.org/, and Epidemiology at http://www.epidem.com/). Information on the STROBE Initiative is available at http://www.strobe-statement.org.

For peer review only

For peer review only - http://bmjopen.bmj.com/site/about/guidelines.xhtml

BMJ Open

Medical negligence claims and the health and life satisfaction of Australian doctors: a prospective cohort analysis of the MABEL survey.

Journal:	*BMJ Open*
Manuscript ID	bmjopen-2021-059447.R1
Article Type:	Original research
Date Submitted by the Author:	20-Apr-2022
Complete List of Authors:	Bradfield, Owen; The University of Melbourne, Melbourne School of Population and Global Health;<br>Bismark, Marie; University of Melbourne, Centre for Health Policy<br>Scott, Anthony; The University of Melbourne, Melbourne Institute of Applied Economic and Social Research<br>Spittal, Matthew; The University of Melbourne, Melbourne School of Population and Global Health
**Primary Subject Heading**:	Health policy
Secondary Subject Heading:	Epidemiology, Public health
Keywords:	MENTAL HEALTH, Health & safety < HEALTH SERVICES ADMINISTRATION & MANAGEMENT, Health policy < HEALTH SERVICES ADMINISTRATION & MANAGEMENT, Quality in health care < HEALTH SERVICES ADMINISTRATION & MANAGEMENT, MEDICAL LAW, PUBLIC HEALTH

SCHOLARONE™
Manuscripts

I, the Submitting Author has the right to grant and does grant on behalf of all authors of the Work (as defined in the below author licence), an exclusive licence and/or a non-exclusive licence for contributions from authors who are: i) UK Crown employees; ii) where BMJ has agreed a CC-BY licence shall apply, and/or iii) in accordance with the terms applicable for US Federal Government officers or employees acting as part of their official duties; on a worldwide, perpetual, irrevocable, royalty-free basis to BMJ Publishing Group Ltd ("BMJ") its licensees and where the relevant Journal is co-owned by BMJ to the co-owners of the Journal, to publish the Work in this journal and any other BMJ products and to exploit all rights, as set out in our licence.

The Submitting Author accepts and understands that any supply made under these terms is made by BMJ to the Submitting Author unless you are acting as an employee on behalf of your employer or a postgraduate student of an affiliated institution which is paying any applicable article publishing charge ("APC") for Open Access articles. Where the Submitting Author wishes to make the Work available on an Open Access basis (and intends to pay the relevant APC), the terms of reuse of such Open Access shall be governed by a Creative Commons licence – details of these licences and which Creative Commons licence will apply to this Work are set out in our licence referred to above.

Other than as permitted in any relevant BMJ Author's Self Archiving Policies, I confirm this Work has not been accepted for publication elsewhere, is not being considered for publication elsewhere and does not duplicate material already published. I confirm all authors consent to publication of this Work and authorise the granting of this licence.

For peer review only

**ARTICLE TYPE**	**Original research**
**TITLE**	**Medical negligence claims and the health and life satisfaction of Australian doctors: a prospective cohort analysis of the MABEL survey.**
**Authors**	Dr Owen Bradfield[1] (owenmb@student.unimelb.edu.au)<br>Prof Marie Bismark[1] (mbismark@unimelb.edu.au)<br>Prof Anthony Scott[2] (a.scott@unimelb.edu.au)<br>Prof Matthew Spittal[3] (m.spittal@unimelb.edu.au)<br><br>[1] Law and Public Health Unit, Centre for Health Policy, Melbourne School of Population and Global Health, University of Melbourne, Australia.<br>[2] Melbourne Institute: Applied Economic and Social Research, Faculty of Business and Economics, University of Melbourne, Australia.<br>[3] Centre for Mental Health, Melbourne School of Population and Global Health, University of Melbourne, Australia.
**Corresponding author**	Dr Owen Bradfield, PhD Candidate, Law and Public Health Unit, Centre for Health Policy, Melbourne School of Population and Global Health, University of Melbourne, 207 Bouverie Street, Carlton, VIC 3053, Australia.<br>Email: owenmb@student.unimelb.edu.au.<br>ORCID ID: 0000-0002-8955-7432
**Word count**	**3602**

For peer review only

ABSTRACT

Objective: To assess the association between medical negligence claims and doctors' self-rated health and life satisfaction.

Design: Prospective cohort study

Participants: Registered doctors practising in Australia who participated in waves 4 to 11 of the Medicine in Australia: Balancing Employment and Life (MABEL) longitudinal survey between 2011 and 2018.

Primary and secondary outcome measures: Self-rated health and self-rated life satisfaction.

Results: Of the 15,105 doctors in the study, 885 reported being named in a medical negligence claim. Fixed-effects linear regression analysis showed that both self-rated health and self-rated life satisfaction declined for all doctors over the course of the MABEL survey, with no association between wave and being sued. However, being sued was not associated with any additional declines in self-rated health (coef. = -0.01, 95% CI -0.05 to 0.02, p=0.41) or self-rated life satisfaction (coef. = -0.01, 95% CI -0.08 to 0.06, p=0.83) after controlling for a range of job factors. Instead, we found that working conditions and job satisfaction were the strongest predictors of self-rated health and self-rated life satisfaction in sued doctors. In analyses restricted to doctors who were sued, we observed no changes in self-rated health (p=0.99) or self-rated life satisfaction (p=0.59) in the years immediately following a claim.

Conclusions: In contrast to prior overseas cross-sectional survey studies, we show that medical negligence claims do not adversely affect the wellbeing of doctors in Australia when adjusting for time trends and previously established covariates. This may be because: 1) prior studies failed to adequately address issues of causation and confounding; 2) legal processes governing medical negligence claims in Australia cause less distress compared to those in other jurisdictions. Our findings suggest that the interaction between medical negligence claims and poor doctors' health is more complex than revealed through previous studies.

Abstract word count: 293

For peer review only

Article summary:

Strengths and limitations of our study

- The key strength of this study is that it we employed a longitudinal design and adjusted for relevant covariates when assessing whether experiencing a medical negligence claim is associated with a change in self-rated health and self-rated life satisfaction.
- The use of seven years of cohort data, and the ability to adjust for demographic, vocational, and psychosocial factors that contribute to poor doctor health, enabled clearer causal inferences to be drawn between medical negligence claims and psychosocial outcomes.
- Doctors were lost to follow up at the end of each wave, which may have resulted in a selection bias in that doctors with poorer health may have been less likely to remain in successive waves of the survey.
- Data on exposure and outcome variables were based on self-report, as official statistics from courts or insurers on the number of doctors sued is not publicly available in Australia.
- Only a small proportion of doctors participating in the survey were sued, which might have reduced our capacity to detect a statistically significant difference in self-rated health and life satisfaction between sued doctors and controls.

Acknowledgements: This research used data from the MABEL longitudinal survey of doctors. MABEL was developed by the Melbourne Institute of Applied Economic and Social Research and Monash University, Melbourne, Australia. Funding for MABEL was provided by the National Health and Medical Research Council (2007 to 2016: 454799 and 1019605); the Australian Government Department of Health and Ageing (2008); Health Workforce Australia (2013); and in 2017 The University of Melbourne, Medibank Better Health Foundation, the NSW Ministry of Health, and the Victorian Department of Health and Human Services. In 2018, funding was provided by the Australian Government Department of Health.

Funding statement: Dr Owen Bradfield was funded by the Australian Government on a 2020 Melbourne Research Scholarship and is a 2020 Fulbright Future Scholar. Prof Marie Bismark was funded by the Australian Government on a National Health and Medical Research Council

For peer review only

Investigator Grant (APP1195984). Prof Matthew Spittal was funded by the Australian Government on an Australian Research Council Future Fellowship (FT180100075).

Competing interests: None

Provenance and peer review: Not commissioned; externally peer reviewed.

Data sharing statement: De-identified MABEL survey data are available upon application to the Australian Data Archive.

Contributorship statement:

OB and MS developed the initial idea and methodology for the study and undertook the statistical analyses. They are also jointly responsible for the overall data as guarantors. AS provided technical expertise about the MABEL protocol and variables. MB assisted with the interpretation of results from a patient safety and doctors' health perspective. MB and AS revised the draft critically for important intellectual content. All authors have given final approval for the article to be published.

For peer review only

Introduction

Medical negligence litigation allows patients who suffer injury or loss resulting from substandard medical care to seek redress through the legal system.[1] However, the system has been criticised for being slow, expensive, inaccessible,[2] and emotionally distressing for patients and doctors.[3] This is a societal problem,[4] as accumulating research shows that poor doctor wellbeing adversely affects doctors' clinical performance and decision-making, which can directly affect the quality of care that patients receive.[5]

Prior cross-sectional studies in the UK,[6] Ireland,[7] US,[8] and Canada[9] have converged on the conclusion that medical negligence litigation adversely impacts doctors' health. For example, a recent self-report survey of nearly 8000 UK doctors found that doctors with a current or recent claim were approximately twice as likely as doctors without a claim to report suicidal ideation or moderate-severe depression and anxiety. Another study showed that doctors who were sued experienced higher rates of depression, anxiety, post-traumatic stress disorder, and suicidal ideation than doctors who were not.[10] Doctors who were sued identified the litigation process as their most stressful life experience: more so than divorce or the death of a spouse.[11] Even doctors who have not been sued identified the 'threat of litigation' as their most serious work-related stressor[12] and this was associated with doctors leaving the profession.[13]

The principal limitation of this prior body of research is that it almost entirely relies on cross-sectional surveys[14] that cannot easily disentangle the temporal relationship between the causes and consequences of medical negligence claims. This limits the ability to infer a causal association and means, for instance, that it is not possible to reliably untangle whether being sued increases the risk of poor health or whether poor health increases the risk of being sued, or both. It also limits the extent to which known risk factors for poor doctor health, including high job demands, low job control, and low work-life balance can be considered as confounders or alternative explanations of any exposure-outcome association. These issues could be resolved using prospective cohort data because individuals can be followed over time, allowing observation of outcomes before and after a claim. It would also allow the impact of confounders on the exposure-outcome association to be observed.

In this study, we used the Medicine in Australia: Balancing Employment and Life (MABEL) cohort to assess the relationship between self-reported medical negligence claims and self-rated

For peer review only

health and self-rated life satisfaction. We used a fixed-effects regression approach to control for unobserved stable individual factors as well as observed time-varying factors. Based on prior studies, we hypothesised that, compared to doctors who were not sued, doctors who were sued would experience poorer psychological outcomes when controlling for confounding factors (measures of job quality). We further hypothesised that among doctors who were sued, there would be a decline in psychological outcomes in the year of a claim and in the year or two following a claim when compared to their outcomes prior to being sued. Our primary outcome measure was self-rated health, and our secondary outcome measure was self-rated life satisfaction.

Methods

Data source

MABEL is a longitudinal panel survey of doctors' working conditions, job satisfaction, work-life balance, health, and life satisfaction. It comprised 11 annual waves that were collected between 2008 and 2018. The initial cohort was drawn from a national directory of 59,620 practicing doctors in Australia, with new medical graduates and newly registered overseas trained doctors invited to participate in subsequent waves. These new recruits replaced doctors lost to follow-up and maintained the cross-sectional representativeness of the cohort. MABEL excluded doctors who had retired, were on leave, or working overseas. MABEL was developed by researchers at the Melbourne Institute of Applied Economic and Social Research and Monash University, Melbourne, Australia. Copies of the survey instruments are publicly available[15] and a detailed description of the MABEL protocol and cohort has been published elsewhere.[16]

Sample selection

We restricted our analysis to survey responses from waves four to 11 (2011 to 2018) because earlier waves did not include questions about medical negligence claims. We excluded doctors-in-training and hospital non-specialists because they are usually hospital employees. In Australia, hospitals are vicariously liable for the negligence of doctors within their employ.[17] This means that the hospital, rather than its employed doctors, would defend medical any negligence litigation.

Variables of interest

For peer review only

Our primary outcome was self-rated health. The survey question was "In general, would you say your health is: excellent, very good, good, fair, poor". We recoded this variable so that higher scores indicated better health. This tool is a strong predictor of mortality[18] and has been used in other large health research surveys.[19] Our secondary outcome was self-rated life satisfaction. This was measured using a 10-point scale asking respondents to self-rate their life satisfaction from 1 ("completely dissatisfied") to 10 ("completely satisfied"). Higher scores are indicative of greater satisfaction. Surveys of life satisfaction have been shown to be stable and sensitive to changing life circumstances.[20]

Our key exposure variable was being a defendant in a medical negligence claim. This was constructed by combining responses to two questions: "In the preceding 12 months have you been named in a medical negligence claim?" (yes or no); and "how long ago did it happen?" (≤3 months, 4-6 months, 7-9 months, 10-12 months ago). Responses were coded 0 for all waves prior to being sued and 1 from the wave the respondent reported being sued onwards. Doctors who were not sued during the study period were coded 0 throughout.

To adjust for the potential confounding effect of job satisfaction, we constructed four variables which we included in our models: high job demands, low job control, poor social supports, and work-life imbalance. These four variables were derived from the "Job satisfaction" questions contained in the MABEL survey, which themselves were drawn from the Warr-Cook-Wall Job Satisfaction Scale,[21] and have been validated for use in the Australian medical workforce context.[22] Previous research has shown that higher scores on these four variables are associated with higher odds of poorer self-rated health.[23]

High job demands was measured using four items scored 0 to 4 (strongly disagree to strongly agree). These were "It is difficult to take time off when I want to", "My patients have unrealistic expectations about how I can help them", "Running my practice is stressful most of the time" and "The majority of my patients have complex health and social problems". Low job control was measured using five items: "Freedom to choose your own method of working?" (scored from 0 "very dissatisfied" to 4 "very satisfied", reverse coded); "Amount of variety in your work?" (scored from 0 "very dissatisfied" to 4 "very satisfied", reverse coded); "Amount of responsibility you are given" (scored from 0 "very dissatisfied" to 4 "very satisfied", reverse coded); "The hours I work are predictable" (scored from 0 "strongly disagree" to 4 "strongly agree"); and "I am restricted in my employment and/or the time and hours I work due to lack

For peer review only

of available childcare" (scored from 0 "strongly disagree" to 4 "strongly agree"). Poor social supports were measured using three items scored 0 to 4 (strongly disagree to strongly agree). These were: "I have a poor support network of other doctors like me"; "I don't have many friends or family members in my current work location"; and "It is easy to pursue my hobbies and leisure interests in my current work location" (reverse coded). Work-life balance was measured using four items: "The balance between my personal and professional commitments is about right" (scored from 0 "strongly disagree" to 4 "strongly agree"), "My hours of work" (scored from 0 "very dissatisfied" to 4 "very satisfied"), "I can take time off at short notice, for example if one of my children is ill or for a home emergency" (scored from 0 "strongly disagree" to 4 "strongly agree") and "My colleagues understand the need for work-life balance" (scored from 0 "strongly disagree" to 4 "strongly agree"). High job demands, low job control, poor social support and work-life balance were all converted to z scores with means of 0 and standard deviations of 1.

We also adjusted for the potential confounding effect of age and hours worked per week. Age at each wave was coded into 5-year bands (<35, 35-39, 40-44, 45-49, 50-54, 55-59, 60-64, 65-69, ≥70 years). Working hours was coded into three categories based on the Australian Bureau of Statistics' definition of a standard full-time working week.[24] These were <35 hours per week, 35 to 45 hours per weeks and >45 hours per week.

We also had information on the following time-invariant variables that we set to their baseline values: sex (male, female); speciality (general practitioners, adult medicine physicians, surgeons, paediatricians, anaesthetists, pathologists and radiologists, emergency physicians, obstetricians and gynaecologists, ophthalmologists, psychiatrists, other); dependent children (collapsed into "none" or "one or more"); currently living with partner or spouse (yes or no); and geographical location (collapsed into three categories based on the five-category Australian Standard Geographical Classification[25]).

Statistical analyses

We first described the characteristics of the cohort at baseline using counts and percentages. We then performed fixed-effects linear regression analyses to examine changes in self-rated health and self-rated life satisfaction. Fixed effects models are useful in the context of cohort data because they estimate the average within-person change in the outcome according to

For peer review only

time-varying covariates entered into the model. Our key predictor was the binary variable coded 0 prior to being sued and 1 from the wave a doctor reported being sued onwards. Doctors who were never sued were coded 0 throughout. We adjusted for wave, hours worked, high job demands, low job control, poor social supports, work-life balance, and age. We also performed post hoc tests to determine if there was an interaction (effect modification) between being sued and wave. Finally, to explore a possible temporal relationship between being sued and the outcomes, we undertook an analysis only among those doctors who reported being sued. Our key predictor was a variable that distinguished between the years prior to being sued, the year they were sued, one year after being sued, two years after being sued, and so on. Thus, we used fixed effects methods to determine if self-rated health and satisfaction with life changed after doctors were sued, and if so, how long any change persisted for. Our models included the same set of covariates as the main models. All analyses were conducted using Stata 16.1 (StataCorp, College Station, TX, USA).

Ethics

The MABEL survey was approved by The University of Melbourne Faculty of Business and Economics Human Ethics Advisory Group (Ref. 0709559) and the Monash University Standing Committee on Ethics in Research Involving Humans (Ref. CF07/1102 -2007000291). Our study was approved by the Melbourne School of Population and Global Health Human Ethics Advisory Group (Ref. 1956096). The Melbourne Institute: Applied Economic and Social Research granted access to de-identified MABEL survey responses. Participant consent for publication was not required.

Patient and public involvement

It was not possible to involve patients or the public in the design, conduct, reporting, or dissemination plans of our research. Results will be made available to MABEL participants at https://melbourneinstitute.unimelb.edu.au/mabel/results-and-publications/journal-articles.

Results

Demographic details of survey participants

Between 2011 and 2018, 15,105 doctors were available for analysis. 885 (5.90%) reported being sued at least once. The characteristics of doctors included for analysis are shown in Table

For peer review only

1. They were predominately male (56%), general practitioners (53%), working more than 45 hours per week (28%), and practicing in metropolitan areas (70%). The majority were living with their spouse or partner (85%) and had at least one dependent child (61%). The baseline mean score for self-rated health was 3.07 (SD = 0.92, range 0 to 4), where the maximum score represented excellent health. The mean self-rated life satisfaction score was 7.42 (SD = 1.62, range 1 to 10) where the maximum score represented complete satisfaction. Doctors were followed for between 1 and 8 waves, with a mean of 3.8 waves. One third of doctors (n = 5,847) completed 5 or more of the 8 waves of data collection. 18% of doctors reported being sued in the first wave (2011) and 7% reported being sued in the last wave (2018). The remaining 75% reported being sued in the intervening waves with the proportion sued for the first time declining in each wave.

Association between being sued and self-rated health and life satisfaction

Multivariate fixed-effects linear regression analysis indicated that mean self-rated health declined each wave (coef. = -0.04, 95% CI -0.05 to -0.04, p < 0.001) (Table 2). There was no evidence that being sued was associated with any additional declines in self-rated health (coef. = -0.02, 95% CI -0.06 to 0.02, p = 0.39). High job demands (coef. = -0.02 per 1 standard deviation [SD] increase in scores, 95% CI -0.03 to -0.01, p < 0.001), low job control (coef. = -0.04 per SD increase, 95% CI -0.05 to -0.03, p<0.001), and poor social supports (coef. = -0.03 per SD increase, 95% CI -0.04 to -0.02, p<0.001) were all associated with lower self-rated health. Achieving work-life balance (coef. = 0.04 per SD increase, 95% CI 0.03 to 0.05, p < 0.001) was associated with higher self-rated health. There was no evidence that hours worked per week (p = 0.21) or age (p = 0.14) were associated with self-rated health. In a post hoc test there was no evidence of an interaction between wave and being sued (p = 0.13).

A similar set of findings emerged for self-rated life satisfaction. Mean life satisfaction declined during each wave of data collection (coef = -0.06, 95% CI -0.07 to -0.05, p<0.001) and being sued was not associated with any further decline (coef. = -0.01, 95% CI -0.08 to 0.07, p = 0.91). Results for all other predictors were similar, as detailed in Table 2, and in a post hoc test there was no evidence of an interaction between wave and being sued (p = 0.42).

Temporal association between being sued and self-rated health and life satisfaction

Among doctors who had been sued, we found no evidence that self-rated health or self-rated life satisfaction changed in the years after a claim was made (Table 3). Compared to a sued

For peer review only

doctors' self-rated health in the years prior to a claim, there was no evidence that their health changed in the year of a claim (coef. = -0.01. 95% CI -0.07 to 0.06), the year after the claim (coef. = -0.01, 95% CI -0.09 to 0.07) or any of the other subsequent years. The same pattern of results was repeated for life-satisfaction.

Discussion

Doctors' health has been described as a "global health-care predicament", with emerging evidence that poor doctor wellbeing adversely affects healthcare quality and safety.[26] Doctors who are unwell take more time off work, leading to workforce understaffing, increased staff turnover, and increased healthcare expenditure.[27] Similarly, studies have linked poor doctor health with suboptimal patient care and a doubling of the risk of medical errors.[28] Factors contributing to poor doctor health include: professional stressors (long working hours, shift work, workplace violence); a blame culture in medicine; fear and stigma of discussing health concerns with colleagues; and easy access to medications that leads to self-prescribing.[29] There is also mounting research suggesting that medico-legal claims and complaints may contribute to poor doctor health.[30]

Providing good patient care is central to doctors' professional identities. Doctors are notoriously self-critical and interpret allegations made against them during litigation as an assault on their professional competence and integrity,[31] which can lead to re-traumatisation[32] and vocational disenchantment.[33] Litigation can also inflict financial and reputational damage,[34] while fear of future litigation can lead to defensive behaviours such as over-investigation or avoidance of high-risk patients and procedures.[35] These effects may be compounded by legal advice that discourages doctors from speaking about their litigation experience with colleagues and peers for fear of compromising their claim or breaching confidential settlement terms.[36]

However, in contrast to previous research, our study did not find an association between medical negligence claims and self-rated health and self-rated life satisfaction. There are possible several explanations for this. First, prior studies adopted a cross-sectional design, which means that causation and the impact of time and other confounders could not be verified. Second, prior studies often examined the association between doctor health and various types of medico-legal claims, such as complaints or regulatory investigations, rather than medical

For peer review only

negligence litigation specifically. Complaints and regulatory investigations may affect doctors more severely, or in different ways, than litigation. Third, the legal processes and frameworks governing medical negligence claims differ between jurisdictions. Those processes in Australia may cause less distress than processes overseas.

In Australia, tort law reforms enacted 20 years ago aimed at curtailing medical negligence litigation may have positively impacted the litigation experience for doctors in Australia.[37] Following widespread concerns that the volume and cost of medical litigation was making medical indemnity insurance unaffordable and unavailable for many doctors,[38] reforms were introduced that included: i) shortening time-limits within which proceedings may be initiated; ii) limiting claims to "significant" injuries; iii) capping compensation payments; and iv) mandating mediation.[39] It is also compulsory for Australian doctors to have professional indemnity insurance.[40] As a result of these reforms, there are now fewer medical negligence claims against doctors. In our study, the proportion of doctors who reported being sued for the first time declined year on year. Of those medical negligence claims that are commenced, the overwhelming majority settle out of court on confidential terms. This may mean that Australian medical negligence claims are rarely subject to media scrutiny and are less likely to inflict financial or reputational damage on doctors. In addition, medical defence insurers and legal practitioners play a crucial role in supporting and educating sued doctors about the personal and professional impacts of legal processes.[41] Our results may also suggest that sued doctors in Australia are better supported professionally and personally, compared to overseas.

Nevertheless, despite the absence of a correlation between medical negligence claims and poor doctor health, our findings add weight to growing calls to improve doctors' health. Self-rated health and self-rated life-satisfaction declined on average throughout the duration of the study. Growing demands on doctors, associated with higher patient expectations, and increased administrative and regulatory requirements, may have contributed to this finding. We found that high job demands, low job control, poor social support, and a work-life balance were all associated with self-rated health and life satisfaction. This is consistent with previous findings that showed an association between poor psychosocial working conditions and self-rated health in doctors.[42] It is likely that workplace factors have significantly contributed to declining doctor health over the seven years studied. This reinforces the pressing need for ongoing efforts to support doctors' health and wellbeing, particularly during the pandemic. Doctors are often ashamed to disclose to peers that they are unwell for fear of being judged. Being unable to

For peer review only

share their experiences may exacerbate feelings of isolation. As a group, unwell doctors are often silent and invisible with few available avenues of peer support. This needs to change, as prior research shows that doctors enjoy better psychological wellbeing when supported by family, colleagues, or employers.[43]

Our study had several limitations. First, a number of doctors were lost to follow up at the end of each wave, which may have resulted in a selection bias in that doctors with poorer health may have been less likely to remain in successive waves of the survey. Second, data in relation to exposure to a medical negligence claim and the primary and secondary health and life satisfaction outcomes were self-reported, as official statistics from courts or insurers on the number of doctors sued is not publicly available in Australia. Third, as only a small proportion of doctors participating in the survey were sued, we were unable to detect a statistically significant difference in self-rated health and life satisfaction between sued doctors and controls. In a *post hoc* power calculation, we estimated that we had 13% power to detect the observed difference of -0.02 between sued and non-sued doctors on self-rated health. However, this difference is very close to zero, and the explanation that there is no association between being sued and self-rated health or life-satisfaction after adjustment for time seems more likely than the explanation that the study lacked power. This is because an indication of lack of power would be a large effect size with wide confidence intervals that included the null value; whereas we observed small effect sizes close to zero. A substantially larger sample of doctors who had been sued would be required to detect a difference between groups of this magnitude. Despite these limitations, the key strengths of this study were: its large sample size; the prospective cohort study design that enabled us to draw stronger causal inferences than previous studies; the assessment of a wide range of demographic, vocational, and psychosocial covariables; and controlling for time-invariant bias within and between persons.

While there are reports of doctors who have died by suicide in the context of medical regulatory investigations,[44] our large longitudinal analysis of doctors in Australia found no association between medical negligence claims and poor doctors' health. This may be because medical negligence claims have less impact on doctors compared to regulatory complaints or investigations. It may also be because any adverse impact of claims on doctors' health is short-lived and does not translate into lower self-rated health or life satisfaction scores 12 months later. Instead, we found a significant association between workplace factors and doctors' health, suggesting that workplace health and safety reforms, rather than further tort law

For peer review only

reforms, ought to be a priority for continued work in improving the health and wellbeing of doctors and thereby benefitting the patients they serve.

For peer review only

Table 1: Characteristics of doctors at baseline (n = 15,105)

Characteristic	n	%
Sex		
Female	6,718	44.5
Male	8,334	55.2
Missing	53	0.4
Age group		
<35	2,387	15.8
35-39	2,376	15.7
40-44	2,117	14.0
45-49	1,873	12.4
50-54	1,832	12.1
55-59	1,585	10.5
60-64	1,105	7.3
65-69	702	4.6
≥70	634	4.2
Missing	494	3.3
Speciality		
General practitioner	7,539	49.9
Adult medicine	1,760	11.7
Surgery	686	4.5
Paediatrics	427	2.8
Anaesthesia	1,007	6.7
Pathology and radiology	437	2.9
Emergency	403	2.7
Obstetrics and gynaecology	393	2.6
Ophthalmology	181	1.2
Psychiatry	634	4.2
Dermatology	68	0.5
Other	612	4.1
Missing	958	6.3
Work location		
Metropolitan	10,097	66.8
Regional/Rural	2,775	18.4
Remote	1,515	10.0
Missing	718	4.8
Hours worked per week		
<35	5,081	33.6
35-45	5,149	34.1
>45	4,182	27.7
Missing	693	4.6
Dependent children		
None	5,196	34.4
One or more	8,318	55.1
Missing	1,591	10.5
Living with a spouse or partner		
Yes	11,623	76.9
No	2,036	13.5
Missing	1,446	9.6

For peer review only

Table 2: Fixed effects regression predicting self-rated health and satisfaction with life

	**Self-rated health (51,099 observations among 13,841 doctors)**		**Satisfaction with life (51,119 observations among 13,821 doctors)**	
	Coefficient (95% CI)	p value	Coefficient (95% CI)	p value
**Medical negligence claim**		0.40		0.91
No	Ref.		Ref.	
Yes	-0.02 (-0.06 to 0.02)		-0.01 (-0.08 to 0.07)	
**Wave** (per 1 wave increase)	-0.04 (-0.05 to -0.04)	<0.001	-0.06 (-0.07 to -0.05)	<0.001
**Hour of work per week**		0.22		0.034
<35 hours	Ref.		Ref.	
35-45 hours	0.02 (-0.01 to 0.03)		0.03 (-0.01 to 0.06)	
>45 hours	0.02 (-0.01 to 0.04)		-0.01 (-0.06 to 0.03)	
**High job demands** (per 1 SD increase)	-0.02 (-0.03 to -0.01)	<0.001	-0.11 (-0.12 to -0.09)	<0.001
**Low job control** (per 1 SD increase)	-0.04 (-0.05 to -0.03)	<0.001	-0.25 (-0.27 to -0.24)	<0.001
**Poor social supports** (per 1 SD increase)	-0.03 (-0.04 to -0.02)	<0.001	-0.15 (-0.16 to -0.13)	<0.001
**Work life balance** (per 1 SD increase)	0.04 (0.03 to 0.05)	<0.001	0.21 (0.19 to 0.22)	<0.001
**Age**		0.14		<0.001
≤35 years	Ref.		Ref.	
35-39 years	-0.03 (-0.07 to 0.01)		0.01 (-0.07 to 0.09)	
40-44 years	-0.05 (-0.10 to 0.01)		-0.07 (-0.17 to 0.03)	
45-49 years	-0.04 (-0.10 to 0.03)		-0.18 (-0.30 to -0.06)	
50-54 years	-0.03 (-0.10 to 0.05)		-0.23 (-0.37 to -0.09)	
55-59 years	-0.03 (-0.12 to 0.05)		-0.21 (-0.37 to -0.05)	
60-64 years	-0.01 (-0.11 to 0.09)		-0.17 (-0.35 to 0.01)	
65-69 years	0.03 (-0.08 to 0.14)		-0.06 (-0.26 to 0.15)	
≥70 years	0.04 (-0.09 to 0.17)		0.01 (-0.24 to 0.25)	

For peer review only

Table 3: Effect of time since claim on self-rated health or life satisfaction in doctors who were sued

	**Self-rated health (4,615 observations among 882 doctors)**		**Self-rated life satisfaction (4,713 observations among 885 doctors)**	
	**Coefficient (95% CI)**	**p value**	**Coefficient (95% CI)**	**p value**
**Time since claim**		0.99		0.82
Years prior to a claim	Ref.		Ref.	
Year of claim	-0.01 (-0.07 to 0.06)		-0.07 (-0.19 to 0.05)	
One year after claim	-0.01 (-0.09 to 0.07)		-0.03 (-0.18 to 0.12)	
Two years after a claim	0.01 (-0.08 to 0.10)		-0.07 (-0.24 to 0.11)	
Three years after a claim	-0.01 (-0.11 to 0.10)		-0.11 (-0.31 to 0.10)	
Four or more years after a claim	-0.02 (-0.15 to 0.11)		-0.08 (-0.33 to 0.17)	
**Wave** (per 1 wave increase)	-0.05 (-0.07 to -0.03)	<0.001	-0.05 (-0.08 to -0.02)	0.006
**Hour of work per week**		0.11		0.009
<35 hours	Ref.		Ref.	
35-45 hours	0.02 (-0.04 to 0.08)		0.12 (0.01 to 0.24)	
>45 hours	0.07 (-0.01 to 0.14)		0.21 (0.08 to 0.35)	
**High job demands** (per 1 SD increase)	-0.01 (-0.04 to 0.02)	0.68	-0.08 (-0.14 to -0.02)	0.006
**Low job control** (per 1 SD increase)	-0.05 (-0.07 to -0.02)	0.001	-0.34 (-0.39 to -0.29)	<0.001
**Poor social supports** (per 1 SD increase)	-0.04 (-0.07 to -0.02)	0.001	-0.22 (-0.27 to -0.17)	<0.001
**Work life balance** (per 1 SD increase)	0.04 (0.01 to 0.07)	0.003	0.24 (0.19 to 0.29)	<0.001
**Age**		0.041		0.012
≤35 years	Ref.		Ref.	
35-39 years	-0.21 (-0.38 to 0.04)		0.25 (-0.58 to 0.07)	
40-44 years	-0.23 (-0.44 to 0.02)		-0.49 (-0.89 to -0.09)	
45-49 years	-0.10 (-0.34 to 0.15)		-0.52 (-0.99 to -0.05)	
50-54 years	-0.07 (-0.34 to 0.21)		-0.54 (-1.07 to -0.01)	
55-59 years	-0.03 (-0.34 to 0.28)		-0.43 (-1.02 to 0.17)	
60-64 years	0.05 (-0.30 to 0.39)		-0.39 (-1.05 to 0.27)	
65-69 years	0.07 (-0.31 to 0.45)		-0.07 (-0.81 to 0.66)	
≥70 years	0.08 (-0.35 to 0.51)		-0.01 (-0.84 to 0.82)	

For peer review only

References

[1] Vincent C, Young M, Phillips A. Why do people sue doctors? A study of patients and relatives taking legal action. *Lancet*. 1994;343(8913):1609-13. doi: 10.1016/s0140-6736(94)93062-7. PMID: 7911925.

[2] Bielen S, Grajzl P, Marneffe W. The resolution process and the timing of settlement of medical malpractice claims. *Health Econ Policy Law*. 2020;15(4):509-529. doi: 10.1017/S1744133119000185. Epub 2019 Apr 17. PMID: 30994084.

[3] Nash L, Curtis B, Walton M, Willcock S, Tennant C. The response of doctors to a formal complaint. *Australas Psychiatry*. 2006 Sep;14(3):246-50. doi: 10.1080/j.1440-1665.2006.02295.x. PMID: 16923032.

[4] Keet M, Heavin H, Sparrow S. Anticipating and managing the psychological cost of civil litigation. *Windsor Yearbook of Access to Justice/Recueil annuel de Windsor d'accès à la justice*. 2017;34(2):73-98, at 85.

[5] Wallace JE, Lemaire JB, Ghali WA. Physician wellness: a missing quality indicator. *Lancet*. 2009 Nov 14;374(9702):1714-21. doi: 10.1016/S0140-6736(09)61424-0. PMID: 19914516.

[6] Bourne T, Wynants L, Peters M, Van Audenhove C, Timmerman D, Van Calster B, Jalmbrant M. The impact of complaints procedures on the welfare, health and clinical practise of 7926 doctors in the UK: a cross-sectional survey. *BMJ Open*. 2015 Jan 15;5(1):e006687. doi: 10.1136/bmjopen-2014-006687. PMID: 25592686; PMCID: PMC4316558.

[7] Tumelty, M. Exploring the emotional burdens and impact of medical negligence litigation on the plaintiff and medical practitioner: Insights from Ireland. *Legal Studies,* 2021; 1-24. doi:10.1017/lst.2021.20.

[8] Balch CM, Oreskovich MR, Dyrbye LN, Colaiano JM, Satele DV, Sloan JA, Shanafelt TD. Personal consequences of malpractice lawsuits on American surgeons. *J Am Coll Surg*. 2011 Nov;213(5):657-67. doi: 10.1016/j.jamcollsurg.2011.08.005. Epub 2011 Sep 3. PMID: 21890381.

[9] Cook WR, Neff C. Attitudes of physicians in northern Ontario to medical malpractice litigation. *Can Fam Physician*. 1994; 40:689-98. PMID: 8199521; PMCID: PMC2380112.

[10] Jain A, Ogden J. General practitioners' experiences of patients' complaints: qualitative study. *BMJ*. 1999 Jun 12;318(7198):1596-9. doi: 10.1136/bmj.318.7198.1596. PMID: 10364121; PMCID: PMC28140.

[11] Charles SC, Warnecke RB, Nelson A, Pyskoty CE. Appraisal of the event as a factor in coping with malpractice litigation. *Behav Med*. 1988 Winter;14(4):148-55. doi: 10.1080/08964289.1988.9935139. PMID: 3256367.

[12] Schattner PL, Coman GJ. The stress of metropolitan general practice. *Med J Aust*. 1998 Aug 3;169(3):133-7. doi: 10.5694/j.1326-5377.1998.tb116013.x. PMID: 9734508.

[13] Charles SC, Wilbert JR, Franke KJ. Sued and nonsued physicians' self-reported reactions to malpractice litigation. *Am J Psychiatry*. 1985 Apr;142(4):437-40. doi: 10.1176/ajp.142.4.437. PMID: 3976916.

[14] Nash LM, Kelly PJ, Daly MG, Walter G, van Ekert EH, Walton M, Willcock SM, Tennant CC. Australian doctors' involvement in medicolegal matters: a cross-sectional self-report study. *Med J Aust*. 2009 Oct 19;191(8):436-40. doi: 10.5694/j.1326-5377.2009.tb02879.x. PMID: 19835537.

[15] Medicine in Australia: Balancing Employment and Life. University of Melbourne. Available at: https://melbourneinstitute.unimelb.edu.au/mabel (accessed 29 June 2021).

[16] Joyce CM, Scott A, Jeon S-H, Humphreys J, Kalb G, Witt J, Leahy A. The "Medicine in Australia: Balancing Employment and Life (MABEL)" longitudinal survey--protocol and baseline data for a prospective cohort study of Australian doctors' workforce participation. *BMC Health Serv Res* 2010; 10(1): 1–10. doi: 10.1186/1472-6963-10-50. PMID: 20181288; PMCID: PMC2837653.

[17] White B, McDonald FJ, Willmott L. *Health law in Australia*. Lawbook Company; 2014: 375.

[18] Idler EL, Benyamini Y. Self-rated health and mortality: a review of twenty-seven community studies. J Health Soc Behav. 1997 Mar;38(1):21-37. PMID: 9097506.

[19] Subramanian SV, Huijts T, Avendano M. Self-reported health assessments in the 2002 World Health Survey: how do they correlate with education?. *Bulletin of the World Health Organization*. 2010; 88:131-8.

[20] Siahpush M, Spittal M, Singh GK. Happiness and life satisfaction prospectively predict self-rated health, physical health, and the presence of limiting, long-term health conditions. *Am J Health Promot*. 2008;23(1):18-26. doi: 10.4278/ajhp.061023137. PMID: 18785370.

[21] Warr P, Cook J, Wall T. Scales for the measurement of some work attitudes and aspects of psychological well-being. *Journal of Occupational Psychology*. 1979;52(2):129-48.

[22] Hills D, Joyce C, Humphreys J. Validation of a job satisfaction scale in the Australian clinical medical workforce. *Eval Health Prof*. 2012;35(1):47-76. doi: 10.1177/0163278710397339. PMID: 21411473.

[23] Milner A, Witt K, Spittal MJ, Bismark M, Graham M, LaMontagne AD. The relationship between working conditions and self-rated health among medical doctors: evidence from seven waves of the Medicine In Australia Balancing Employment and Life (Mabel) survey. *BMC Health Serv Res*. 2017 Aug 29;17(1):609. doi: 10.1186/s12913-017-2554-z. PMID: 28851354; PMCID: PMC5576303.

[24] ABS. Underemployed Workers, Australia, September 2013. Cat. No. 6265.0. Canberra: Australian Bureau of Statistics; 2013.

[25] Australian Bureau of Statistics (2006) *Australian Standard Geographical Classification (ASGC)*. Australian Bureau of Statistics, Canberra. Available at: https://www.abs.gov.au/websitedbs/d3310114.nsf/home/australian+standard+geographical+classification+(asgc). Accessed 2 July 2021.

[26] The Lancet. Physician burnout: the need to rehumanise health systems. *Lancet*. 2019 Nov 2;394(10209):1591. doi: 10.1016/S0140-6736(19)32669-8. PMID: 31690430.

[27] Carayon P, Cassel C, Dzau VJ. Improving the System to Support Clinician Well-being and Provide Better Patient Care. *JAMA*. 2019 Dec 10;322(22):2165-2166. doi: 10.1001/jama.2019.17406. PMID: 31644783.

[28] Fahrenkopf AM, Sectish TC, Barger LK, et al. Rates of medication errors among depressed and burnt out residents: prospective cohort study. *BMJ* 2008; 336(7642): 488-91. doi: 10.1136/bmj.39469.763218.BE. Epub 2008 Feb 7. PMID: 18258931; PMCID: PMC2258399.

[29] National Academies of Sciences, Engineering, and Medicine; National Academy of Medicine; Committee on Systems Approaches to Improve Patient Care by Supporting Clinician Well-Being. Taking Action Against Clinician Burnout: A Systems Approach to Professional Well-Being. Washington (DC): National Academies Press (US); 2019 Oct 23. PMID: 31940160.

[30] Browne P, Haysom G. Supportive networks, healthier doctors and 'just culture': Managing the effects of medico-legal complaints on doctors. *Aust J Gen Pract*. 2019 Jan-Feb;48(1-2):9-12. doi: 10.31128/AJGP-09-18-4713. PMID: 31256451.

[31] American College of Obstetricians and Gynecologists. ACOG Committee opinion no. 551: coping with the stress of medical professional liability litigation. *Obstet Gynecol*. 2013; 121(1): 220-2. doi: 10.1097/01.aog.0000425665.64635.1c. PMID: 23262965.

For peer review only

[32] Scott SD, Hirschinger LE, Cox KR, McCoig M, Brandt J, Hall LW. The natural history of recovery for the healthcare provider "second victim" after adverse patient events. *Qual Saf Health Care*. 2009;18(5):325-30. doi: 10.1136/qshc.2009.032870. PMID: 19812092.

[33] Debono B, Hamel O, Guillain A, Durand A, Rué M, Sabatier P, Lonjon G, Dran G; French Society of Private Neurosurgeons (SFNCL). Impact of malpractice liability among spine surgeons: A national survey of French private neurosurgeons. *Neurochirurgie*. 2020 Aug;66(4):219-224. doi: 10.1016/j.neuchi.2020.05.003. Epub 2020 Jun 12. PMID: 32540341.

[34] Nash L, Tennant C, Walton M. The psychological impact of complaints and negligence suits on doctors. *Australas Psychiatry*. 2004 Sep;12(3):278-81; discussion 282. doi: 10.1080/j.1039-8562.2004.02079.x. PMID: 15715791.

[35] Reisch LM, Carney PA, Oster NV, Weaver DL, Nelson HD, Frederick PD, Elmore JG. Medical malpractice concerns and defensive medicine: a nationwide survey of breast pathologists. *Am J Clin Pathol*. 2015 Dec;144(6):916-22. doi: 10.1309/AJCP80LYIMOOUJIF. PMID: 26572999; PMCID: PMC6436915.

[36] Eisenberg RL. Reactions of physicians sued for malpractice. In: Radiology and the Law 2004 (pp. 74-77). Springer, New York, NY.

[37] Panel of Eminent Persons to Review the Law of Negligence (the Ipp Committee), *Review of the Law of Negligence, Final Report.* Canberra, October 2002. Available at: https://treasury.gov.au/sites/default/files/2019-03/R2002-001_Law_Neg_Final.pdf (accessed 16 July 2021)

[38] Luntz, H. (2002). Reform of the Law of Negligence: Wrong Questions - Wrong Answers. *UNSW Law Journal*, 25(3), 836–841. doi:10.3316/informit.119529917834518.

[39] Skene L. Law and medical practice: rights, duties, claims and defences. 3rd ed. Australia: LexisNexisButterworths, 2008.

[40] *Health Practitioner Regulation National Law Act 2009* (Qld), section 129.

[41] Haysom G. The impact of complaints on doctors. *Aust Fam Physician*. 2016;45(4):242-4. PMID: 27052144.

[42] Elovainio M, Salo P, Jokela M, Heponiemi T, Linna A, Virtanen M, Oksanen T, Kivimäki M, Vahtera J. Psychosocial factors and well-being among Finnish GPs and specialists: a 10-year follow-up. *Occup Environ Med*. 2013 Apr;70(4):246-51. doi: 10.1136/oemed-2012-100996. Epub 2013 Jan 15. PMID: 23322916.

[43] Bourne T, De Cock B, Wynants L, Peters M, Van Audenhove C, Timmerman D, Van Calster B, Jalmbrant M. Doctors' perception of support and the processes involved in complaints investigations and how these relate to welfare and defensive practice: a cross-sectional survey of the UK physicians. *BMJ Open*. 2017 Nov 21;7(11):e017856. doi: 10.1136/bmjopen-2017-017856. PMID: 29162574; PMCID: PMC5719304.

[44] Horsfall S. *Doctors who commit suicide while under GMC fitness to practice investigation* (GMC Report, 14 December 2014) < https://www.gmc-uk.org/-/media/documents/Internal_review_into_suicide_in_FTP_processes.pdf_59088696.pdf> (accessed 8 November 2021).

For peer review only

STROBE Statement—Checklist of items that should be included in reports of ***cohort studies***

	Item No	Recommendation	Page No
**Title and abstract**	1	(*a*) Indicate the study's design with a commonly used term in the title or the abstract	1
		(*b*) Provide in the abstract an informative and balanced summary of what was done and what was found	1
**Introduction**			
Background/rationale	2	Explain the scientific background and rationale for the investigation being reported	2
Objectives	3	State specific objectives, including any prespecified hypotheses	2
**Methods**			
Study design	4	Present key elements of study design early in the paper	3
Setting	5	Describe the setting, locations, and relevant dates, including periods of recruitment, exposure, follow-up, and data collection	3-6
Participants	6	(*a*) Give the eligibility criteria, and the sources and methods of selection of participants. Describe methods of follow-up	3
		(*b*) For matched studies, give matching criteria and number of exposed and unexposed	n/a
Variables	7	Clearly define all outcomes, exposures, predictors, potential confounders, and effect modifiers. Give diagnostic criteria, if applicable	4-5
Data sources/ measurement	8*	For each variable of interest, give sources of data and details of methods of assessment (measurement). Describe comparability of assessment methods if there is more than one group	4-5
Bias	9	Describe any efforts to address potential sources of bias	n/a
Study size	10	Explain how the study size was arrived at	6
Quantitative variables	11	Explain how quantitative variables were handled in the analyses. If applicable, describe which groupings were chosen and why	3-6
Statistical methods	12	(*a*) Describe all statistical methods, including those used to control for confounding	5
		(*b*) Describe any methods used to examine subgroups and interactions	
		(*c*) Explain how missing data were addressed	
		(*d*) If applicable, explain how loss to follow-up was addressed	
		(*e*) Describe any sensitivity analyses	
**Results**			
Participants	13*	(a) Report numbers of individuals at each stage of study—eg numbers potentially eligible, examined for eligibility, confirmed eligible, included in the study, completing follow-up, and analysed	5-6
		(b) Give reasons for non-participation at each stage	
		(c) Consider use of a flow diagram	
Descriptive data	14*	(a) Give characteristics of study participants (eg demographic, clinical, social) and information on exposures and potential confounders	6-7
		(b) Indicate number of participants with missing data for each variable of interest	6-7
		(c) Summarise follow-up time (eg, average and total amount)	6-7
Outcome data	15*	Report numbers of outcome events or summary measures over time	7-8

For peer review only - http://bmjopen.bmj.com/site/about/guidelines.xhtml

Main results	16	(*a*) Give unadjusted estimates and, if applicable, confounder-adjusted estimates and their precision (eg, 95% confidence interval). Make clear which confounders were adjusted for and why they were included<br>(*b*) Report category boundaries when continuous variables were categorized<br>(*c*) If relevant, consider translating estimates of relative risk into absolute risk for a meaningful time period	8
Other analyses	17	Report other analyses done—eg analyses of subgroups and interactions, and sensitivity analyses	n/a
**Discussion**			
Key results	18	Summarise key results with reference to study objectives	8-10
Limitations	19	Discuss limitations of the study, taking into account sources of potential bias or imprecision. Discuss both direction and magnitude of any potential bias	10
Interpretation	20	Give a cautious overall interpretation of results considering objectives, limitations, multiplicity of analyses, results from similar studies, and other relevant evidence	8-10
Generalisability	21	Discuss the generalisability (external validity) of the study results	8-10
**Other information**			
Funding	22	Give the source of funding and the role of the funders for the present study and, if applicable, for the original study on which the present article is based	See title page

*Give information separately for exposed and unexposed groups.

Note: An Explanation and Elaboration article discusses each checklist item and gives methodological background and published examples of transparent reporting. The STROBE checklist is best used in conjunction with this article (freely available on the Web sites of PLoS Medicine at http://www.plosmedicine.org/, Annals of Internal Medicine at http://www.annals.org/, and Epidemiology at http://www.epidem.com/). Information on the STROBE Initiative is available at http://www.strobe-statement.org.

For peer review only
